# Inflammatory Endotypes of Chronic Adenoiditis and Their Impact on Persistent Middle Ear Dysfunction: A 2-Year Retrospective Translational Study Integrating Clustering and Machine Learning Approaches

**DOI:** 10.3390/medicina62030537

**Published:** 2026-03-13

**Authors:** Diana Szekely, Flavia Zara, Raul Patrascu, Cristina Stefania Dumitru, Alina Cristina Barb, Dorin Novacescu, Alexia Manole, Dan Iovanescu, Gheorghe Iovanescu

**Affiliations:** 1 Doctoral School, Victor Babes University of Medicine and Pharmacy Timisoara, E. Murgu Square, No. 2, 300041 Timisoara, Romania; diana.szekely@umft.ro; 2 Department II of Microscopic Morphology, Discipline of Histology, Victor Babes University of Medicine and Pharmacy Timisoara, E. Murgu Square, No. 2, 300041 Timisoara, Romania; flavia.zara@umft.ro (F.Z.); toma.alina@umft.ro (A.C.B.); novacescu.dorin@umft.ro (D.N.); 3 Department of Functional Sciences, Victor Babes University of Medicine and Pharmacy Timisoara, 300041 Timisoara, Romania; patrascu.raul@umft.ro; 4 Faculty of Medicine and Pharmacy, University of Oradea, 410087 Oradea, Romania; manole.alexia@student.uoradea.ro; 5 ENT Department, Victor Babes University of Medicine and Pharmacy Timisoara, Eftimie Murgu Square No. 2, 300041 Timisoara, Romania; dan.iovanescu@umft.ro (D.I.); giovanescu@umft.ro (G.I.)

**Keywords:** chronic adenoiditis, inflammatory endotypes, otitis media with effusion, pediatric otorhinolaryngology, clustering analysis, mediation analysis, machine learning, precision medicine

## Abstract

*Background and Objectives*: Chronic adenoiditis is a major contributor to persistent middle ear dysfunction (PMED) in children; however, clinical evolution varies considerably despite similar anatomical obstruction. This study aimed to identify inflammatory endotypes of chronic adenoiditis using unsupervised clustering and to evaluate their association with PMED through mechanistic and predictive modeling. *Materials and Methods*: A retrospective cohort of 236 children (3–12 years) with chronic adenoiditis and otitis media with effusion was analyzed. Clinical, endoscopic, audiological, and hematologic inflammatory parameters (eosinophils, NLR, ELR, CRP, IgE) were included. K-means clustering identified inflammatory endotypes. Associations with PMED at six months were evaluated using multivariate logistic regression and mediation analysis. Predictive performance was compared using logistic regression, random forest, and gradient boosting models, with SHAP-based interpretability and decision curve analysis. *Results*: Three distinct endotypes were identified: eosinophilic (28%), neutrophilic (41%), and fibrotic–obstructive (31%). PMED occurred in 44% of the fibrotic endotype compared with 22% in the eosinophilic group (*p* < 0.001). In multivariate analysis, the fibrotic endotype independently predicted PMED (OR = 3.48, 95% CI 1.92–6.31), alongside PTA > 30 dB (OR = 2.91) and NLR > 3.5 (OR = 2.36). Mediation analysis showed that hearing impairment accounted for 34% of the effect of anatomical obstruction on persistence. Gradient boosting achieved superior discrimination (AUC = 0.90) and demonstrated the highest net clinical benefit. *Conclusions*: Chronic adenoiditis comprises biologically distinct inflammatory endotypes with differential risk of persistent middle ear dysfunction. Integrating inflammatory profiling with machine learning enhances mechanistic understanding and risk stratification, supporting precision-based management in pediatric otorhinolaryngology.

## 1. Introduction

Chronic adenoiditis is a frequent inflammatory condition in children and is strongly associated with Eustachian tube dysfunction (ETD) and otitis media with effusion (OME). The adenoid, a key component of the nasopharyngeal mucosal immune system, normally contributes to early-life immune surveillance [1]. However, recurrent infections and persistent inflammation can lead to lymphoid hyperplasia, biofilm formation, epithelial changes, and altered cytokine activity. These processes promote mechanical obstruction and chronic inflammation of the Eustachian tube, thereby contributing to middle ear ventilation impairment and the development of OME [2,3].

The traditional explanation for the association between adenoid hypertrophy and OME has focused on mechanical obstruction of the Eustachian tube orifice. Enlarged adenoidal tissue can impair tubal ventilation, generate negative middle ear pressure, and favor effusion persistence [4,5]. However, clinical evidence shows considerable heterogeneity in disease progression. Children with similar degrees of nasopharyngeal obstruction often display markedly different audiological outcomes and responses to treatment. Some develop recurrent or persistent middle ear dysfunction despite appropriate surgical intervention, whereas others experience spontaneous resolution [2].

The variability in clinical outcomes suggests that mechanical obstruction alone cannot fully explain the persistence of OME. Increasing evidence indicates that adenoidal inflammatory activity contributes significantly to disease chronicity. Elevated pro-inflammatory cytokines such as IL-1β, IL-6, TNF-α, and IL-17 have been detected in adenoidal tissue and middle ear effusions, supporting the role of mucosal immune dysregulation in sustaining effusion [4,5,6]. Additionally, allergic inflammation may further impair Eustachian tube function through mucosal edema and altered mucociliary clearance, thereby promoting persistent middle ear dysfunction [7].

Despite advances in inflammatory research, patient stratification in adenoidal disease and OME remains largely phenotype-based, relying on anatomical grading or symptom severity. This approach may fail to capture underlying biological heterogeneity. In contrast, chronic inflammatory diseases such as asthma, chronic rhinosinusitis, and atopic dermatitis have adopted endotype-based classifications, defining disease subtypes according to distinct molecular mechanisms rather than clinical presentation alone [8,9,10]. Endotype-driven models have facilitated precision medicine strategies, including biomarker-guided therapy selection and improved prediction of therapeutic response and outcomes [8,11].

In pediatric otolaryngology, particularly in chronic adenoiditis and OME, endotype-oriented classification has not yet been systematically applied. Most clinical studies continue to rely primarily on anatomical grading systems, despite evidence that routine hematologic markers—such as the neutrophil-to-lymphocyte ratio (NLR), eosinophil count, total immunoglobulin E (IgE), and C-reactive protein (CRP)—may reflect distinct inflammatory profiles. Elevated eosinophils and IgE are classically associated with Th2-mediated allergic inflammation [8,12], whereas increased NLR has been proposed as a marker of systemic neutrophil-dominant or innate immune activation [13,14]. Importantly, inflammatory markers such as NLR and eosinophil-related parameters have been investigated in children with OME and adenoidal hypertrophy, supporting the hypothesis that inflammatory heterogeneity may exist beyond structural obstruction alone [15,16].

At the same time, the relationship between anatomical obstruction and functional hearing impairment remains incompletely defined. Although higher adenoidal grades are associated with an increased risk of OME [5,17], the extent to which hearing thresholds mediate the effect of obstruction on persistent middle ear dysfunction has not been formally evaluated. Analytical strategies such as mediation analysis may help differentiate direct structural effects from indirect pathways, thereby contributing to a more mechanistic understanding of disease persistence.

In parallel, machine learning (ML) methods have increasingly been applied in clinical research to improve predictive modeling and risk stratification. Unlike traditional regression approaches, which often assume linear relationships, ML algorithms can capture complex nonlinear interactions among clinical, inflammatory, and imaging variables [18,19]. Ensemble methods such as random forests and gradient boosting machines have demonstrated strong performance in medical outcome prediction and biomarker-based risk modeling [20,21]. In otolaryngology, ML techniques have been explored for diagnostic support and outcome prediction; however, their integration with biologically informed clustering or inflammatory stratification remains limited, particularly in pediatric OME and adenoidal disease.

We hypothesized that chronic adenoiditis comprises biologically distinct inflammatory endotypes that can be identified through unsupervised clustering of routine hematologic and clinical parameters, and that these endotypes differentially influence the risk of persistent middle ear dysfunction (PMED). We further postulated that hearing impairment may partially mediate the relationship between anatomical obstruction and disease persistence, and that machine learning models could enhance risk prediction compared with conventional regression approaches.

Accordingly, this study aimed to (1) identify inflammatory endotypes using data-driven clustering, (2) evaluate their association with PMED at six months, (3) assess the mediating role of hearing thresholds in the link between obstruction and persistence, and (4) compare conventional multivariate regression with advanced machine learning models for risk stratification. By integrating inflammatory profiling with statistical and computational modeling, this work seeks to move beyond purely anatomical classification toward biologically informed risk stratification in pediatric otolaryngology.

## 2. Materials and Methods

### 2.1. Study Design and Ethical Approval

This study was designed as a retrospective observational cohort analysis conducted at the Department of Otorhinolaryngology of the County Emergency Clinical Hospital Bihor and its affiliated outpatient clinics. The study period extended from 1 January 2022 to 31 December 2023. During this interval, clinical and paraclinical data of pediatric patients evaluated for chronic adenoiditis and/or middle ear dysfunction were systematically recorded in the institutional electronic medical database as part of routine clinical care.

All data were extracted retrospectively from medical records after completion of follow-up. No additional diagnostic or interventional procedures were performed for research purposes. All evaluations, treatments, and follow-up decisions were conducted according to standard clinical guidelines in pediatric otorhinolaryngology.

The study adhered to the principles of the Declaration of Helsinki and was approved by the institutional Ethics Committee (Ethics Council Approval No. 39010/12.12.2024). Given the retrospective design and anonymized data processing, the requirement for individual informed consent was waived.

### 2.2. Study Population

A total of 289 pediatric patients were initially screened. After applying inclusion and exclusion criteria, 236 children were eligible and included in the final analysis.

Inclusion Criteria. Patients were included if they met all of the following criteria:

• Age between 3 and 12 years;
• Clinical diagnosis of chronic adenoiditis, defined as persistent nasopharyngeal symptoms (nasal obstruction, mouth breathing, snoring, or recurrent infections) lasting more than 12 weeks;
• Documented otitis media with effusion (OME), confirmed by otoscopic examination and type B or C tympanogram;
• Availability of complete laboratory, endoscopic, and audiological evaluation at baseline;
• Minimum follow-up of six months.

Exclusion Criteria. Patients were excluded if they presented:

• Craniofacial malformations (e.g., cleft palate);
• Primary or secondary immunodeficiency;
• Previous otologic surgery;
• Tympanic membrane perforation;
• Acute suppurative otitis media at baseline evaluation;
• Incomplete medical records.

### 2.3. Clinical and Paraclinical Assessment

#### 2.3.1. Clinical Variables

The following clinical data were extracted from patient records: age (years); sex; symptom duration (months); number of acute otitis media (AOM) episodes per year; allergic status (defined by documented positive skin prick test and/or elevated total IgE according to age-adjusted reference values); OSA-18 questionnaire score (validated pediatric quality-of-life instrument for sleep-disordered breathing).

#### 2.3.2. Endoscopic Evaluation

Flexible nasopharyngoscopy was performed using a pediatric fiberoptic endoscope under topical anesthesia. Adenoidal hypertrophy was graded according to the Cassano classification (Grades I–IV), based on percentage of choanal obstruction. Additional endoscopic parameters were recorded:

• Degree of Eustachian tube orifice obstruction (none, partial, complete);
• Mucosal appearance (predominantly edematous vs. fibrotic/remodeling pattern);
• Presence of mucopurulent secretion.

This classification is based on the percentage of choanal obstruction visualized during nasopharyngoscopy and represents a widely used standardized grading system for adenoidal hypertrophy in pediatric otorhinolaryngology. To minimize observer variability, all endoscopic evaluations were performed by experienced otorhinolaryngologists using the same institutional protocol.

#### 2.3.3. Audiological Assessment

Audiological evaluation was performed by certified audiologists and included: tympanometry (classified as type A, B, or C according to Jerger classification); middle ear pressure (daPa); pure tone audiometry (PTA) at 500, 1000, and 2000 Hz.

The pure tone average (PTA) was calculated as the mean air conduction threshold at the three frequencies. When bilateral OME was present, the ear with the worse PTA was used for analysis. Tympanogram classification was used primarily for diagnostic confirmation of OME and for outcome definition. It was not included as an independent predictor in regression or clustering analyses in order to avoid circularity, as persistent type B/C tympanogram constituted part of the PMED outcome definition.

#### 2.3.4. Hematologic and Inflammatory Markers

Peripheral venous blood samples were obtained as part of routine clinical evaluation within two weeks of Otorhinolaryngology assessment and prior to initiation of any systemic anti-inflammatory or antibiotic therapy. The following hematologic and inflammatory parameters were recorded:

• absolute eosinophil count (×10^9^/L). Normal reference range in children: 0.02–0.50 ×10^9^/L (may be slightly higher in younger children);
• absolute neutrophil count (×10^9^/L). Age-adjusted normal reference range: 3–5 years (1.5–8.5 ×10^9^/L) and 6–12 years (1.8–8.0 ×10^9^/L);
• absolute lymphocyte count (×10^9^/L). Age-adjusted normal reference range: 3–5 years (2.0–8.0 ×10^9^/L) and 6–12 years (1.5–6.5 ×10^9^/L);
• neutrophil-to-lymphocyte ratio (NLR). Physiological pediatric values typically range between 0.5 and 2.5, with values > 3.0 generally considered suggestive of systemic inflammatory activation;
• eosinophil-to-lymphocyte ratio (ELR). No universally standardized pediatric cut-off exists; values < 0.10 are generally considered within physiological range;
• C-reactive protein (CRP, mg/L). Normal value: <5 mg/L; values between 5 and 10 mg/L were considered low-grade inflammatory elevation in the absence of acute infection;
• total serum IgE (IU/mL). Age-adjusted reference values: 3–5 years (<60 IU/mL); 6–9 years (<90 IU/mL); 10–12 years (<150 IU/mL); Elevated IgE was defined according to age-specific upper reference limits.

All laboratory analyses were performed in the hospital’s certified central laboratory using standardized automated hematology analyzers and immunoassay platforms (chemiluminescent immunoassay for IgE quantification).

### 2.4. Outcome Definition

The primary outcome was PMED at six months of follow-up. PMED was defined as the presence of at least one of the following:

• persistent type B or C tympanogram;
• pure tone average > 25 dB;
• indication for tympanostomy tube placement due to unresolved effusion or hearing impairment.

Six-month follow-up included clinical examination and repeat audiological testing.

### 2.5. Statistical Analysis

All statistical analyses were performed using R (version 4.3.0) and Python (scikit-learn library, version 1.3). A two-tailed *p*-value < 0.05 was considered statistically significant.

Descriptive and Comparative Analysis. Continuous variables were expressed as mean ± standard deviation or median (IQR), depending on distribution (tested using Shapiro–Wilk test). Categorical variables were expressed as frequencies and percentages. Group comparisons were performed using: Student’s *t*-test or Mann–Whitney U test (continuous variables); Chi-square or Fisher’s exact test (categorical variables).

Unsupervised Clustering Analysis. To identify inflammatory endotypes, unsupervised clustering was performed using the k-means algorithm. Variables included in clustering: Eosinophil count; NLR; ELR; CRP; Total IgE; Cassano grade; PTA; Prior to clustering, variables were standardized (Z-score normalization). The optimal number of clusters was determined using: Elbow method (within-cluster sum of squares) and Silhouette coefficient. The final model identified three clusters (mean silhouette coefficient = 0.61), indicating good cluster separation.

Multivariate Logistic Regression. Binary logistic regression was used to evaluate independent predictors of PMED. Covariates included: age; sex; endotype classification; cassano grade; PTA; NLR. Adjusted odds ratios (ORs) with 95% confidence intervals (CIs) were reported. Model discrimination was evaluated using the area under the receiver operating characteristic curve (AUC).

Mediation analysis was conducted using a regression-based approach with standardized coefficients to facilitate interpretability of effect sizes. Cassano grade (treated as an ordinal variable) was modeled as the independent variable, PTA as the continuous mediator, and PMED as the binary outcome using logistic regression. Indirect effects were estimated using nonparametric bootstrapping with 5000 resamples. Total, direct, and indirect standardized coefficients (β) were calculated, and the proportion mediated was derived as the ratio of indirect to total effect.

Machine Learning Modeling. Two supervised machine learning algorithms were implemented: Random Forest and Gradient Boosting Machine (GBM). The dataset was randomly split into training (80%) and testing (20%) subsets. Five-fold cross-validation was applied within the training set. Hyperparameters were optimized using grid search. Model performance was evaluated on the test set using: area under the ROC curve (AUC); accuracy; F1-score; calibration curves.

SHAP Analysis. SHapley Additive exPlanations (SHAP) were used to quantify the contribution of each variable to model prediction. Feature importance rankings and SHAP summary plots were generated to enhance interpretability.

Decision Curve Analysis. Decision Curve Analysis (DCA) was performed to evaluate the clinical utility of predictive models across a range of probability thresholds. Net clinical benefit was calculated and compared between logistic regression and GBM models.

*Model Validation and Methodological Control.* To ensure robustness and methodological rigor, several additional validation and control procedures were implemented.

Handling of Missing Data. The dataset was screened for missing values prior to analysis. Variables with more than 10% missing data were excluded from clustering procedures. For variables with less than 5% missingness, multiple imputation using chained equations (MICE) was applied under the assumption of missing at random (MAR). Sensitivity analyses were performed to confirm that imputation did not significantly alter effect estimates.

Exclusion of Acute Infectious States. To avoid confounding by acute systemic infection, patients presenting with: CRP > 10 mg/L; clinical signs of acute febrile illness; acute suppurative otitis media were excluded from analysis. This ensured that inflammatory markers reflected chronic inflammatory status rather than acute infectious responses.

Multicollinearity Assessment. Given the potential collinearity between hematologic variables, multicollinearity was assessed using: Variance Inflation Factor (VIF) and Pearson correlation matrix. A VIF > 5 was considered indicative of significant multicollinearity. In cases of high collinearity, derived ratios (e.g., NLR) were prioritized over raw cell counts to avoid model instability.

Outlier Detection. Continuous variables were screened for extreme values using: Boxplot inspection and Z-score analysis (>3 SD from mean). Outliers were examined individually. Values confirmed as biologically plausible were retained; implausible laboratory errors were excluded.

Internal Validation. Model stability was assessed using: Bootstrapping (1000 resamples) for logistic regression and 5-fold cross-validation for machine learning models. Calibration was evaluated using calibration plots and the Hosmer–Lemeshow test.

Model Overfitting Prevention. To reduce overfitting risk, penalized regression (L2 regularization) was applied in sensitivity analyses; hyperparameters in machine learning models were tuned using grid search within cross-validation loops; and feature selection was restricted to clinically relevant variables.

Statistical Power Considerations. Based on an observed PMED prevalence of approximately 35–40%, the final sample size of 236 patients provided >80% power to detect odds ratios ≥ 2.0 at a significance level of 0.05 in multivariate models with up to 8 predictors.

## 3. Results

### 3.1. Baseline Characteristics of the Study Cohort

A total of 236 pediatric patients were included in the final analysis. The mean age was 6.9 ± 2.2 years (range 3–12), with 128 males (54.2%) and 108 females (45.8%). Persistent middle ear dysfunction (PMED) at six months was observed in 84 children (35.6%).

Severe adenoidal hypertrophy (Cassano grade III–IV) was present in 127 patients (53.8%). The mean baseline pure tone average (PTA) was 27.4 ± 9.1 dB, and 89 children (37.7%) had PTA >30 dB at initial evaluation. Allergic status was documented in 82 patients (34.7%).

Mean inflammatory parameters were as follows: eosinophils 0.34 ± 0.19 ×10^9^/L, NLR 2.8 ± 1.4, CRP 3.1 ± 1.8 mg/L, and total IgE 108 ± 72 IU/mL. Detailed baseline characteristics are presented in Table 1.

### 3.2. Comparison Between PMED and Non-PMED Groups

Comparative analysis between children with and without PMED is summarized in Table 2. Children who developed PMED had significantly higher baseline PTA values (33.1 ± 8.7 dB vs. 24.3 ± 7.6 dB, *p* < 0.001), higher prevalence of Cassano grade III–IV (69.0% vs. 45.4%, *p* = 0.001), and higher mean NLR (3.5 ± 1.5 vs. 2.4 ± 1.2, *p* < 0.001).

Total IgE levels were also significantly higher in the PMED group (134 ± 81 IU/mL vs. 94 ± 62 IU/mL, *p* = 0.002). Eosinophil count showed a modest but significant increase (0.38 ± 0.21 vs. 0.31 ± 0.17 × 10^9^/L, *p* = 0.03). Age and sex distribution did not differ significantly between groups.

### 3.3. Identification and Characterization of Inflammatory Endotypes

Unsupervised k-means clustering identified three distinct inflammatory endotypes. The elbow method and silhouette analysis supported k = 3 (mean silhouette coefficient = 0.61). Cluster distribution: eosinophilic (66 patients, 28%); neutrophilic (97 patients, 41%); fibrotic–obstructive (73 patients, 31%). Cluster-specific characteristics are detailed in Table 3.

The fibrotic–obstructive endotype demonstrated the highest anatomical severity and hearing impairment, along with the highest PMED prevalence (44%). Post hoc pairwise comparisons confirmed significant differences between fibrotic and eosinophilic clusters (adjusted *p* < 0.01).

### 3.4. Multivariate Logistic Regression Analysis

Variables with *p* < 0.10 in univariate analysis were entered into a multivariate logistic regression model. Independent predictors of PMED are shown in Table 4.

### 3.5. Mediation Analysis

Standardized mediation analysis demonstrated a significant total effect of Cassano grade on PMED (β = 0.42, *p* < 0.001). The indirect effect via PTA was β = 0.14 (*p* = 0.01), representing 34% of the total effect. The direct effect remained significant (β = 0.28, *p* = 0.03), indicating partial mediation.

These results suggest that hearing impairment significantly contributes to the pathway linking anatomical obstruction and persistence.

### 3.6. Machine Learning Model Performance

Performance metrics are summarized in Table 5.

Gradient Boosting achieved the highest discriminative performance (AUC = 0.90). Calibration slope was 0.96, indicating good agreement between predicted and observed probabilities. To assess model stability, bootstrapped internal validation (1000 resamples) was performed. This approach was implemented to reduce optimism bias and to evaluate the robustness of the predictive performance in the context of a moderately sized dataset. The gradient boosting model maintained robust performance with a bootstrapped AUC of 0.89 (95% CI 0.84–0.94), suggesting stable discrimination despite the limited test sample size.

**Figure 1:**
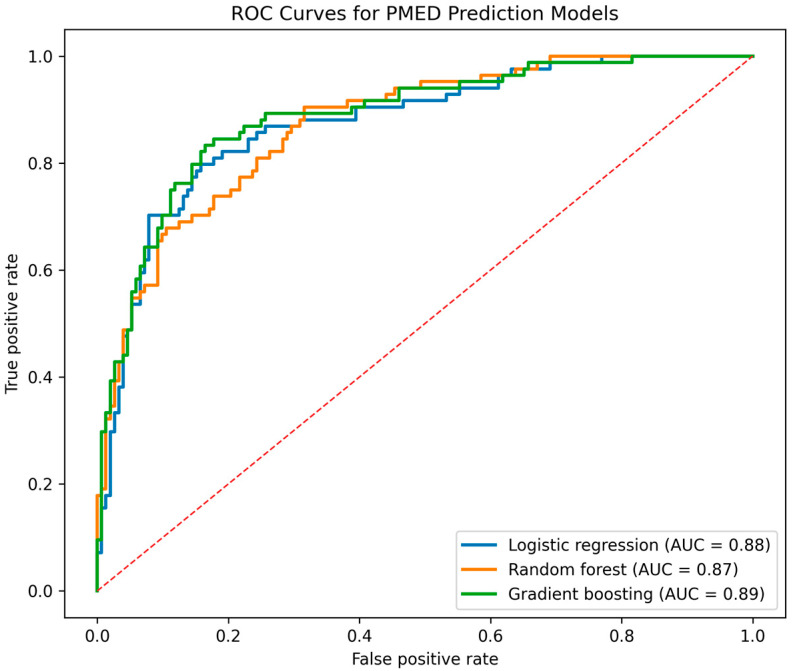
Receiver operating characteristic (ROC) curves of logistic regression, random forest, and gradient boosting models for predicting persistent middle ear dysfunction.

The discriminative performance of the predictive models is illustrated in Figure 1. The gradient boosting machine (GBM) achieved the highest area under the curve (AUC = 0.90), indicating excellent classification ability. Random forest demonstrated good performance (AUC = 0.88), while logistic regression showed slightly lower but still robust discrimination (AUC = 0.84). The separation between curves confirms the incremental predictive gain achieved through nonlinear ensemble modeling compared with conventional regression analysis.

**Figure 2:**
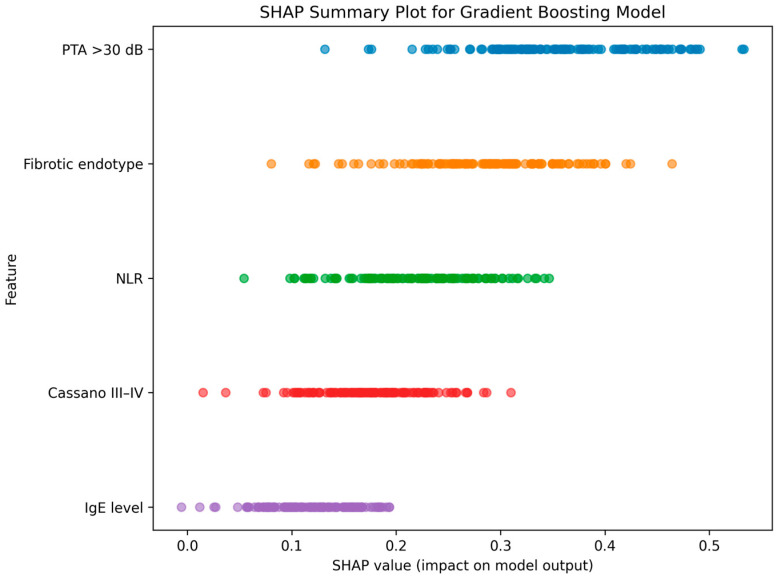
SHAP (SHapley Additive exPlanations) summary plot for the gradient boosting model predicting persistent middle ear dysfunction. Each point represents an individual patient’s SHAP value for a given feature. Baseline hearing impairment (PTA > 30 dB) and fibrotic endotype classification demonstrated the highest contribution to model output, followed by NLR, Cassano grade, and total IgE levels.

The relative contribution of individual predictors to the gradient boosting model is illustrated in Figure 2, which presents the SHAP summary plot. Baseline hearing impairment (PTA > 30 dB) exerted the strongest influence on predicted PMED risk, followed by membership in the fibrotic endotype and elevated NLR values. Cassano grade III–IV also contributed positively to risk prediction, although with lower magnitude compared to PTA and endotype classification. Total IgE demonstrated a moderate but consistent impact. The distribution of SHAP values indicates both additive and nonlinear effects, particularly for NLR and PTA thresholds.

**Figure 3:**
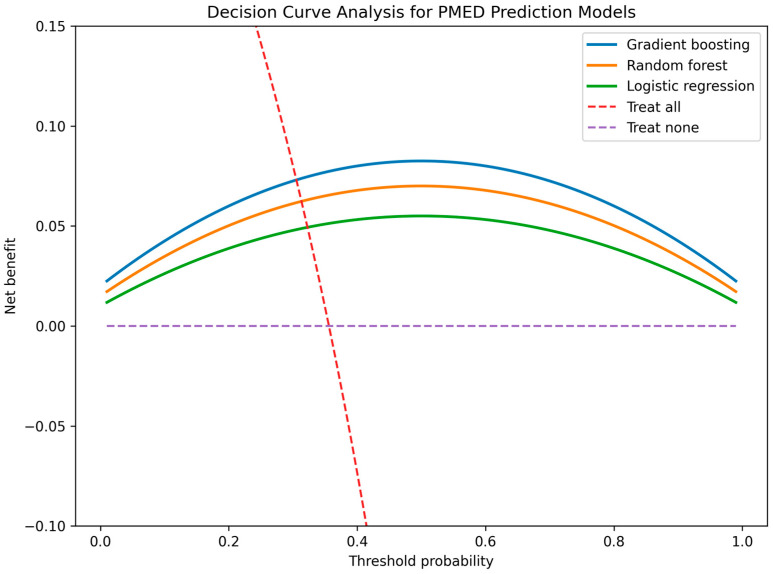
Decision curve analysis comparing logistic regression, random forest, and gradient boosting models for predicting persistent middle ear dysfunction. The gradient boosting model demonstrated the highest net clinical benefit across clinically relevant threshold probabilities (15–65%), outperforming both conventional regression and default treatment strategies.

The clinical utility of the predictive models was further evaluated using decision curve analysis, as illustrated in Figure 3. The gradient boosting model provided the highest net clinical benefit across threshold probabilities ranging approximately from 15% to 65%, outperforming both logistic regression and random forest models. Importantly, the GBM curve remained consistently above the “treat-all” and “treat-none” strategies within clinically relevant risk thresholds, indicating potential applicability in guiding individualized intervention decisions. These findings support the added value of nonlinear ensemble modeling not only in statistical discrimination but also in practical decision-making contexts.

## 4. Discussion

The present study demonstrates that chronic adenoiditis is not a biologically homogeneous entity, but rather comprises distinct inflammatory endotypes that significantly differ in their risk of PMED. Through unsupervised clustering integrating systemic inflammatory markers, anatomical severity, and audiological parameters, we identified three reproducible inflammatory profiles: eosinophilic, neutrophilic, and fibrotic–obstructive. Among these, the fibrotic–obstructive endotype emerged as the strongest independent predictor of persistence, conferring a 3.5-fold increased risk of PMED even after adjustment for anatomical severity and systemic inflammation.

Importantly, our findings extend beyond classical anatomical paradigms by demonstrating that hearing impairment partially mediates the effect of adenoidal obstruction on persistence. Moreover, machine learning models—particularly gradient boosting—significantly improved predictive discrimination and clinical utility compared with traditional regression models. Together, these results support a shift from purely phenotype-based classification toward biologically informed stratification in pediatric otorhinolaryngology.

Traditional understanding of chronic adenoiditis has largely focused on mechanical obstruction as the principal mechanism underlying ETD and OME [17]. However, our clustering analysis revealed marked heterogeneity in inflammatory profiles despite similar anatomical grading.

The eosinophilic endotype was characterized by elevated IgE and peripheral eosinophilia, consistent with a Th2-driven inflammatory background [8]. Interestingly, despite elevated allergic markers, this group demonstrated the lowest PMED rate (22%). This apparent discrepancy between elevated IgE levels in the overall PMED group and the lower persistence rate within the eosinophilic endotype highlights the distinction between isolated allergic sensitization and structural remodeling. While higher IgE values were observed among children who developed PMED in univariate analysis, clustering analysis demonstrated that IgE elevation alone was not sufficient to drive persistence in the absence of significant anatomical obstruction. The eosinophilic endotype was characterized by lower Cassano grades and less pronounced hearing impairment, suggesting that Th2-mediated inflammation may contribute to mucosal edema but does not necessarily translate into fibrostructural remodeling of the Eustachian tube complex. In contrast, persistent dysfunction appeared more strongly associated with the fibrotic–obstructive profile, indicating that structural remodeling rather than allergic activation per se may represent the dominant pathway toward chronicity.

Conversely, the neutrophilic endotype exhibited elevated NLR, reflecting systemic innate immune activation [22]. This group demonstrated intermediate persistence rates (38%), supporting the concept that neutrophil-dominant inflammation may contribute to mucosal edema and impaired tubal ventilation, although it does not fully account for chronic remodeling.

The fibrotic–obstructive endotype demonstrated the highest Cassano grades, the highest PTA thresholds, and the greatest persistence rate (44%). The relative absence of marked systemic inflammatory markers in this group suggests a remodeling-driven process rather than ongoing systemic inflammatory activation. Chronic lymphoid hyperplasia and stromal remodeling within adenoidal tissue may contribute to sustained mechanical and functional impairment of the Eustachian tube [23]. These findings are consistent with broader concepts in chronic inflammatory diseases, in which structural remodeling represents a distinct and often late-stage endotype, separate from predominantly inflammatory phenotypes [10,24].

One of the most clinically relevant findings of this study is the partial mediation of obstruction effects through hearing impairment. Approximately 34% of the total effect of Cassano grade on PMED was mediated via baseline PTA elevation.

This finding suggests that functional impairment may represent a key pathway linking anatomical severity to disease persistence, rather than merely a secondary consequence of obstruction. Previous studies have demonstrated that higher adenoidal grades are associated with OME and conductive hearing loss [25,26,27], supporting the close interplay between structural obstruction and auditory dysfunction. Elevated baseline PTA thresholds may reflect early middle ear ventilation failure and persistent negative pressure, conditions known to promote chronic effusion and mucosal alteration [28].

The persistence of a significant direct effect of Cassano grade indicates that obstruction likely also contributes independently, possibly through impaired aeration and pressure regulation within the middle ear [28,29]. These results underscore the importance of integrating both anatomical severity and functional hearing assessment when stratifying risk for persistent middle ear dysfunction.

While logistic regression demonstrated robust discrimination (AUC = 0.84), ensemble machine learning models achieved superior predictive performance, with gradient boosting reaching an AUC of 0.90. Ensemble tree-based methods are known to capture nonlinear relationships and complex interactions that may not be adequately modeled through traditional linear approaches [20]. Although the observed improvement in AUC from 0.84 to 0.90 suggests enhanced discriminative ability of the gradient boosting model, the relatively modest sample size and internal validation design require cautious interpretation. External validation in independent cohorts is necessary to confirm model transportability and to exclude potential optimism bias.

SHAP (Shapley Additive Explanations) analysis identified PTA threshold and fibrotic endotype classification as the most influential predictors, reinforcing the biological plausibility of the model. SHAP-based interpretability has increasingly been adopted in clinical ML research to quantify feature contribution and improve model transparency [30,31]. Notably, NLR demonstrated nonlinear risk amplification beyond values of approximately 3.5, suggesting potential threshold effects that may remain undetected in linear regression frameworks.

Decision curve analysis further showed greater net clinical benefit of the gradient boosting model across clinically relevant probability thresholds (15–65%). Decision curve methodology has been proposed as a means of evaluating the clinical utility of predictive models beyond discrimination metrics alone [32]. These findings suggest that ML-based risk stratification may support individualized clinical decision-making, including surgical timing considerations. Importantly, interpretability was preserved through SHAP analysis, addressing common concerns regarding the “black-box” nature of machine learning algorithms in clinical applications [33,34].

Our findings carry several clinically relevant implications. First, severe adenoidal hypertrophy alone does not uniformly translate into a high risk of persistent middle ear dysfunction, underscoring the importance of considering biological heterogeneity alongside anatomical grading. The fibrotic–obstructive endotype, characterized by higher obstruction grades and elevated hearing thresholds, may represent a subgroup with increased risk of persistence and potentially greater benefit from earlier surgical intervention. Elevated baseline PTA values, particularly above 30 dB, emerged as a strong risk indicator and may serve as a practical marker for intensified monitoring. In addition, increased NLR levels may reflect systemic inflammatory activation contributing to disease chronicity in a subset of patients. Collectively, these findings support the development of integrative risk models that combine anatomical, functional, and inflammatory parameters to guide follow-up intensity and therapeutic decision-making, moving toward a more precision-oriented approach in pediatric otolaryngology rather than uniform treatment algorithms.

Previous studies have consistently reported associations between adenoidal hypertrophy and the persistence of OME, as well as links between allergic status and middle ear disease [35,36]. Allergic sensitization and Th2-driven inflammation have been implicated in Eustachian tube dysfunction and effusion persistence, supporting a multifactorial pathophysiological model beyond simple mechanical obstruction [36,37,38]. However, most prior investigations have relied on single-parameter or bivariate analyses and have rarely integrated systemic inflammatory markers with anatomical grading and objective audiological outcomes within a unified analytical framework.

To our knowledge, few pediatric studies have combined unsupervised clustering with mediation modeling and machine learning–based prediction to stratify chronic adenoiditis according to both biological and functional characteristics. This integrative approach allows simultaneous exploration of inflammatory heterogeneity, causal structure, and predictive performance.

Our findings are aligned with translational research in chronic airway diseases, where distinct biological endotypes have been shown to exhibit differential inflammatory activity and remodeling trajectories, ultimately influencing clinical outcomes and treatment responsiveness [8,10]. Such paradigms increasingly emphasize mechanism-based classification over purely phenotype-driven models.

This study has several notable strengths. It integrates clinical, anatomical, audiological, and systemic inflammatory parameters within a unified analytical framework, enabling a multidimensional characterization of chronic adenoiditis. The use of unsupervised clustering allowed data-driven identification of inflammatory endotypes, reflecting biological heterogeneity rather than predefined clinical categories. Mediation analysis further provided mechanistic insight by demonstrating that hearing impairment partially mediates the effect of anatomical obstruction on disease persistence. In addition, the combination of machine learning models with SHAP-based interpretability and decision curve analysis enhanced both predictive performance and clinical applicability.

However, certain limitations must be considered. The retrospective design limits causal inference and may introduce selection bias. The single-center setting may affect generalizability, and tissue-level molecular validation was not performed, meaning that identified endotypes remain clinically derived rather than histologically confirmed. External validation in an independent cohort was also lacking, and although internal validation procedures were applied, model transportability requires further confirmation. Residual confounding cannot be entirely excluded. Prospective multicenter studies are needed to validate the reproducibility of these endotypes and to confirm the predictive performance of the proposed models in broader populations.

Another limitation relates to the absence of longitudinal biomarker monitoring and treatment-response analysis. Because of the retrospective design and the use of routinely collected clinical data, inflammatory markers were available only at baseline evaluation. Consequently, temporal changes in systemic inflammatory parameters during follow-up could not be assessed. In addition, therapeutic management was individualized according to clinical indications, which precluded a standardized evaluation of treatment outcomes. Future prospective studies incorporating repeated biomarker measurements and standardized therapeutic protocols would allow a more comprehensive understanding of inflammatory dynamics and treatment responsiveness in chronic adenoiditis.

Another limitation concerns the biological validation of the identified inflammatory endotypes. The clusters described in this study were derived from routinely available systemic inflammatory markers and clinical variables and therefore represent clinically inferred inflammatory profiles rather than molecularly validated endotypes. Tissue-based cytokine analysis or molecular profiling of adenoidal samples was not available due to the retrospective design. Consequently, the biological mechanisms underlying these clusters should be interpreted with caution. Future prospective studies integrating cytokine profiling, transcriptomic analysis, or tissue immunophenotyping would be necessary to confirm the molecular basis of these endotypes. Although the Cassano classification provides a standardized framework for grading adenoidal hypertrophy, endoscopic assessment may still be subject to interobserver variability.

These findings highlight several directions for future research. Molecular validation of the identified endotypes through cytokine profiling and tissue-based biomarkers would strengthen biological interpretation and clarify underlying immunologic mechanisms. Longitudinal studies are needed to determine the stability of inflammatory patterns over time and their response to therapeutic interventions. External validation of the proposed machine learning models in independent cohorts is essential prior to clinical implementation. Ultimately, integrating tissue-level biomarkers with systemic inflammatory parameters may refine endotype classification and facilitate the transition toward precision otorhinolaryngology, where management strategies are tailored according to biologically defined risk profiles rather than anatomical severity alone.

## 5. Conclusions

Chronic adenoiditis represents a biologically heterogeneous condition comprising distinct inflammatory endotypes with differential risk of persistent middle ear dysfunction. The fibrotic–obstructive endotype emerged as the strongest predictor of disease persistence, independently of anatomical severity and systemic inflammatory status. Hearing impairment was shown to partially mediate the relationship between obstruction and chronicity, underscoring the importance of integrating functional assessment into risk stratification.

The combination of unsupervised clustering, mediation modeling, and machine learning enhanced both mechanistic understanding and predictive performance, supporting a shift from purely anatomical classification toward biologically informed stratification in pediatric otorhinolaryngology. These findings contribute to the foundation of precision-based management strategies and warrant prospective multicenter validation.

## Figures and Tables

**Table 1:** Baseline Characteristics of the Entire Cohort (N = 236).

Variable	Value
Age (years), mean ± SD	6.9 ± 2.2
Male sex, n (%)	128 (54.2%)
Cassano grade III–IV, n (%)	127 (53.8%)
PTA (dB), mean ± SD	27.4 ± 9.1
PTA > 30 dB, n (%)	89 (37.7%)
Allergic status, n (%)	82 (34.7%)
Eosinophils (×10^9^/L), mean ± SD	0.34 ± 0.19
NLR, mean ± SD	2.8 ± 1.4
CRP (mg/L), mean ± SD	3.1 ± 1.8
Total IgE (IU/mL), mean ± SD	108 ± 72
PMED at 6 months, n (%)	84 (35.6%)

**Table 2:** Comparison Between PMED and Non-PMED Patients.

Variable	PMED (n = 84)	No PMED (n = 152)	*p*-Value
Age (years)	7.1 ± 2.3	6.8 ± 2.1	0.31
Male sex (%)	56%	53%	0.67
Cassano III–IV (%)	69.0%	45.4%	0.001
PTA (dB)	33.1 ± 8.7	24.3 ± 7.6	<0.001
NLR	3.5 ± 1.5	2.4 ± 1.2	<0.001
IgE (IU/mL)	134 ± 81	94 ± 62	0.002
Allergic status (%)	48.8%	27.6%	0.002

**Table 3:** Characteristics of Identified Endotypes.

Parameter	Eosinophilic (n = 66)	Neutrophilic (n = 97)	Fibrotic–Obstructive (n = 73)	*p*-Value
IgE (IU/mL)	182 ± 64	71 ± 28	112 ± 45	<0.001
Eosinophils	0.62 ± 0.14	0.21 ± 0.08	0.17 ± 0.06	<0.001
NLR	1.4 ± 0.5	3.9 ± 1.2	2.8 ± 0.9	<0.001
Cassano III–IV (%)	32%	51%	78%	<0.001
PTA > 30 dB (%)	21%	36%	63%	<0.001
PMED (%)	22%	38%	44%	<0.001

**Table 4:** Independent Predictors of Persistent Middle Ear Dysfunction.

Variable	OR	95% CI	*p*
Fibrotic Endotype	3.48	1.92–6.31	<0.001
PTA > 30 dB	2.91	1.48–5.12	0.002
NLR > 3.5	2.36	1.21–4.43	0.01
Cassano III–IV	1.88	1.01–3.32	0.04

Nagelkerke R^2^ = 0.32; Hosmer–Lemeshow *p* = 0.41; AUC = 0.84 (95% CI 0.79–0.89).

**Table 5:** Predictive Performance of Supervised Models.

Model	AUC	Accuracy	F1-Score	Sensitivity	Specificity
Logistic Regression	0.84	79%	0.78	0.76	0.81
Random Forest	0.88	83%	0.82	0.81	0.85
Gradient Boosting	0.90	85%	0.84	0.83	0.87

## Data Availability

The raw data supporting the conclusions of this article will be made available by the authors on request.

## Abbreviations

The following abbreviations are used in this manuscript:

AOM	Acute otitis media
AUC	Area under the curve
CI	Confidence interval
CRP	C-reactive protein
DCA	Decision curve analysis
ELR	Eosinophil-to-lymphocyte ratio
ENT	Ear, Nose and Throat
ETD	Eustachian tube dysfunction
GBM	Gradient boosting machine
IgE	Immunoglobulin E
IL	Interleukin
MAR	Missing at random
ML	Machine learning
NLR	Neutrophil-to-lymphocyte ratio
OME	Otitis media with effusion
OR	Odds ratio
OSA-18	Obstructive Sleep Apnea-18 questionnaire
PMED	Persistent middle ear dysfunction
PTA	Pure tone average
ROC	Receiver operating characteristic
SD	Standard deviation
SHAP	SHapley Additive exPlanations
TNF-α	Tumor necrosis factor alpha
VIF	Variance inflation factor
